# Chinese Expert Consensus on the Testing Process, Quality Control and Clinical Application of Fractional Exhaled Nitric Oxide (2025)

**DOI:** 10.1111/crj.70222

**Published:** 2026-09-18

**Authors:** Yulan Qu, Li Li, Jian Guo, Yan Zhou, Ying Gong, Zhihong Chen, Yuanlin Song, Task Force for Pulmonary Function of Chinese Thoracic Society

**Affiliations:** ^1^ Department of Respiratory and Critical Care Medicine Zhongshan Hospital, Fudan University Shanghai China; ^2^ Department of Respiratory Medicine Shanghai Pulmonary Hospital, Tongji University School of Medicine Shanghai China; ^3^ Department of Respiratory Medicine Shanghai General Hospital, Shanghai Jiao Tong University School of Medicine Shanghai China; ^4^ Department of Respiratory Medicine, Xinhua Hospital, Institute of Respiratory Diseases Shanghai Jiao Tong University School of Medicine Shanghai China

**Keywords:** clinical application, fractional exhaled nitric oxide testing, quality control, report, respiratory diseases

## Abstract

Fractional exhaled nitric oxide (FeNO) is an internationally recognized biomarker for type 2 airway inflammation. With the increasing promotion and widespread use of FeNO testing in China, Chinese experts have previously published consensus regarding FeNO measurement principles and clinical applications for adults and children respectively. However, in clinical practice, due to insufficient understanding of the detection principles, quality control requirements, and standardized operational procedures, questions have been raised on FeNO measurement's accuracy and clinical significance, which indeed limit the widespread application of FeNO in clinical settings. Based on the achievements in this area worldwide, this consensus elaborated the details and development on the principle of FeNO instruments, FeNO detection method, operation process, quality control, clinical significance, etc. Hopefully, this consensus document can be used to promote standardized FeNO testing protocols and better serve the precise diagnosis and treatment of airway diseases.

## Graphical abstract

This Chinese expert consensus standardizes fractional exhaled nitric oxide (FeNO) testing—from analyzer principles and detection protocols to quality control and reporting—for reliable measurement of type 2 airway inflammation. It defines clinical cutoffs and delineates the diagnostic and therapeutic roles of FeNO, CaNO, and FnNO across asthma, chronic cough, and upper airway diseases, advocating multi‐parameter integration for precision respiratory care.

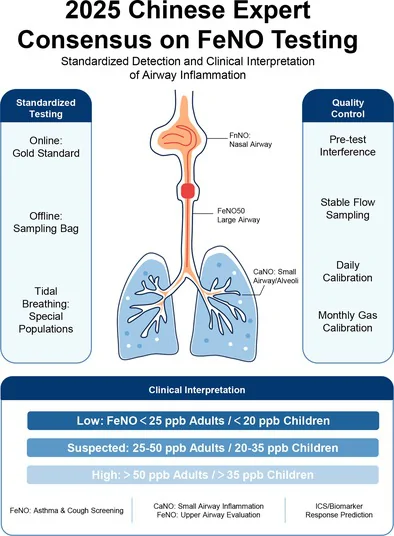

**Abbreviations**
ACQ: Asthma Control Questionnaire
CaNO: concentration of alveolar nitric oxide
CF: cystic fibrosis
ERS: European Respiratory Society
FEF_50_
: forced expiratory flow at 50% of FVC exhaled
FEF_75_
: forced expiratory flow at 75% of FVC exhaled
FeNO: fractional exhaled nitric oxide
FeNO200: fractional exhaled nitric oxide measured at 200 mL/s
FeNO50: fractional exhaled nitric oxide measured at 50 mL/s
FnNO: fractional nasal nitric oxide
ICS: inhaled corticosteroids
NO: nitric oxide
PCD: primary ciliary dyskinesia
PRISMA: Preferred Reporting Items for Systematic Reviews and Meta‐Analyses

The type of inflammation driven by cytokines secreted by T helper 2 (Th2) cells is collectively referred to as type 2 inflammation. FeNO is an internationally recognized biomarker of type 2 airway inflammation, which not only reflects the level of airway inflammation but also predicts the therapeutic effects of inhaled corticosteroids (ICS) and monoclonal antibodies targeting type 2 inflammation. It also has the advantages of being non‐invasive and convenient, making it a common indicator for respiratory diseases diagnosis and follow up.

With the growing adoption of FeNO detection, clinical observations have revealed that a single FeNO measurement may not adequately represent the level of inflammation throughout the entire airway. In 2017, the European Respiratory Society (ERS) recommended standardizing FeNO measurements, including alveolar exhaled nitric oxide (CaNO), oral exhaled nitric oxide (FeNO measured at a flow rate of 200 mL/s), and upper airway nitric oxide (fractional nasally exhaled nitric oxide, or FnNO). This standardization has enhanced the comprehensiveness of FeNO detection.

In China, there are established technical standards for measuring FeNO in children, as well as guidelines for its clinical application. However, expert consensus on FeNO for adults primarily addresses the diagnosis and treatment of airway diseases, lacking comprehensive guidelines on the principles, quality control, and detection processes for FeNO analyzers. Ensuring quality control and standardization in testing is crucial for the accurate interpretation of FeNO data and for informed clinical decision‐making.

In light of this, the Task Force for Pulmonary Function of the Chinese Thoracic Society, alongside relevant experts, has synthesized a series of questions addressing the technical standards and clinical applications of FeNO measurement. This effort, informed by existing consensus statements, guidelines, and recent research, culminates in a consensus document presented in a question‐and‐answer format. The goal is to facilitate the appropriate use of FeNO testing in clinical practice and to guide precise treatment for airway inflammatory diseases.

## 1 Methods and Processes of Consensus Formation

### 1.1 Task Force Composition and Initiation of Consensus‐Setting Tasks

The consensus was initiated by the Task Force for Pulmonary Function of the Chinese Thoracic Society. The panel consisted of respiratory physicians, pulmonary function specialists, allergists, pediatric pulmonologists, and otolaryngologists with extensive experience in FeNO testing and airway inflammatory diseases.

The expert panel included senior academic leaders, experienced clinicians, and early‐career specialists to ensure representation of diverse perspectives and clinical settings. Members were selected based on academic achievements, clinical expertise, and experience in the application of FeNO testing in respiratory diseases.

The consensus was developed by the Task Force for Pulmonary Function of the Chinese Thoracic Society. The expert group reviewed and discussed the evidence related to the principle, quality control, testing process and clinical application of the FeNO analyzer, and formed a consensus draft based on the existing clinical evidence and the collective experience of the working group's members. Based on the consensus reached by working group members through full consultation, the relevant recommendations were revised, and the ultimate consensus was finally formed.

### 1.2 Registration

The consensus has been registered on the International Practice Guidelines Registry and Transparency Platform (http://www.guidelines‐registry.org) (No: PREPARE‐2024CN685).

### 1.3 Literature Search and Evidence Inclusion

The literature search was conducted according to a predefined protocol. The following electronic databases were systematically searched from inception to January 31, 2024:

- PubMed
- Embase
- Web of Science
- CNKI
- Wanfang Database
- Weipu Chinese journal service platform
- China Biomedical Literature Database

The primary search terms included:

(“fractional exhaled nitric oxide” OR “FeNO” OR “exhaled nitric oxide”)

AND

(“working principle” OR “quality control” OR “testing process” OR “clinical application” OR “report”)

Relevant clinical studies, guidelines, consensus statements, systematic reviews, and meta‐analyses were included. Additional references were identified through manual searches of reference lists and key publications. The detailed search strategy and study selection process are presented as a PRISMA flow diagram in Figure S1.

### 1.4 Problem Design and Recommendation Formation

Based on literature retrieval and clinical needs, the consensus expert group designed 21 questions related to the principle, quality control, testing process and clinical application of the FeNO analyzer, sent them to the experts for voting, collected the voting results of each question, and requested each expert to provide evidence supporting their answers, and finally determined 14 questions. After the questions were finalized, a preliminary recommendation was formed through expert discussion. A modified Delphi method was used, involving two rounds of correspondence and one face‐to‐face discussion to form the final recommendation. Seventy‐one experts with extensive FeNO clinical experience were selected nationwide to conduct the Delphi survey in March 2024. The Delphi questionnaire and a summary of evidence from previous literature searches were sent to the members of the expert group by email. Expert group members integrated literature evidence with clinical experience, and considered the pros and cons of recommendations, resource utilization, patient preference, health economics and other factors to fill in the Delphi questionnaire. All consensus questions were voted on using three options: “Agree”, “Neutral”, and “Disagree”. Expert consensus was calculated based on voting results (i.e., the proportion of experts who choose “agree” and “neutral”). If ≥ 90% of experts selected “agree” or “neutral” means that the item is recommended by consensus. The expert group summarized the results of the Delphi survey, distributed 71 questionnaires, and 70 questionnaires were returned, with a 98.6% response rate; 14 questions and recommendations achieved consensus, and this consensus was finally formed.

### 1.5 External Reviews of Consensus

After the first draft of consensus was formed, it was submitted via email to the external review team. The expert group discussed feedback from external reviewers and finally revised and determined the consensus based on the discussion opinions.

In this paper, if the FeNO flow rate is not clearly indicated, the default is 50 mL/s flow rate exhaled nitric oxide (FeNO_50_). If the FnNO does not have a clear flow rate, the default is 10 mL/s of nasal exhaled nitric oxide (FnNO_10_).

## 2 The Structure and Operating Principle of the FeNO Analyzer

**Question (Q) 1:** What are the commonly used FeNO analyzer detection principles? Is there consistency between FeNO analyzers with different detection principles?

**Consensus (C) 1:** There are two main detection principles of FeNO analyzer commonly used in clinical practice: chemiluminescence method and electrochemical method. Chemiluminescence, the first to be applied, is the standard technique. FeNO analyzers with different principles are based on the same technical standards, and some head‐to‐head comparative studies exist domestically and internationally, but there is no consistent conclusion.

The evidence is summarized and interpreted below.

### 2.1 Detection Principle

The chemiluminescence method produces electromagnetic radiation emitted in the form of photons by transiently reacting the NO sampled from the patient's mouth and nose with the ozone generated in the device [1], and the concentration of NO in the sample can be indirectly measured because the emitted light intensity is proportional to the NO concentration in the sampled gas [2]. The response time of its detection is 0.5~0.7 s, the detection sensitivity is 0.1~0.5 ppb (parts per billion, 10^−9^ mol/mol in this paper), it can measure very low‐concentration NO samples with high sensitivity and specificity [3]. However, chemiluminescence analyzers are relatively expensive to operate and maintain, limiting their application.

The electrochemical method uses a current sensor to detect the current generated by the chemical reaction between the sampled gas and the active sensing material [1]. Based on the principle of current detection to determine the concentration of NO in the sample, the performance of the commercially available electrochemical products can reach: response time < 10 s, analysis time < 40 s, detection sensitivity 1.0 ppb with an accuracy of ±2.5 ppb or ±5.0% of the measured value. The electrochemical analyzer is small in size, weighing < 1 kg, simple and portable, economical, and widely used in clinical practice.

### 2.2 Consistency in Instrument Testing Reference Standards

The instrument testing procedures of different principles refer to the technical standards of ATS/ERS [3], including the quality control requirements of inhalation air source, inspiratory phase, exhalation phase, and interfering gas in the exhaled breath of the human body, etc., excluding potential errors from operation or sample itself in each sampling process, and theoretically the samples collected by each instrument and their detection values should be consistent [3].

### 2.3 Variations in Instrument Interference Factors, Technical Parameters, and Design Details

Chemiluminescence analyzers are sensitive to temperature and humidity, sunlight exposure, volatile anesthetic gases, ethanol‐containing disinfectants, etc. [4]. Electrochemical analyzers are affected by a variety of interfering substances, such as nitrogen dioxide, hydrogen sulfide, isoprene, acetonitrile, acetaldehyde, acetone, carbon dioxide, ethanol, and hydrogen peroxide. When these interfering substances are present in the breath sample, differences may occur between devices.

Technical parameters vary slightly across devices and the detection sensitivity range is 0.1~10.0 ppb with repeatability < 3%–10%. The detection range varies in size: range includes 0.1~5000 ppb and 5~300 ppb. This may cause measurement variations near the detection limits. From a clinical point of view, it is recommended that the device should meet at least the following performance requirements: the detection range is 5~300 ppb (if FnNO is detected, the detection range is 5~3000 ppb), and the accuracy is ±5.0 ppb or ±10% of the measured value.

There are also differences in the design details of different devices. FnNO concentration is flow‐dependent, and if the device is equipped with an automatic flow modulation module, it can help the expiratory flow to be closer to the target flow rate, and promote more consistent test results [4]. Depending on the filter used by the device, there may be differences in the fit and tightness of the mask or buccal filter, and therefore potential differences in breath sampling.

## 3 Flow Dependence of FeNO Detection and Its Associated Detection Parameters

### 3.1 Q2: Is FeNO Detection Flow‐Dependent? How Does Expiratory Flow Affect FeNO Testing?

**C2**: Maintaining a stable expiratory flow is important for testing NO at different parts of the airway, and FeNO testing is flow‐dependent. The expiratory flow is 50 ± 5 mL/s for FeNO_50_ and (200 ± 20) mL/s for FeNO_200_. When actually measured, expiratory flow exceeding this range results in a lower FeNO value and vice versa.

The evidence is summarized and interpreted below.

#### 3.1.1 FeNO Values for Different Expiratory Flows and the Proposal of a Two‐Chamber Model

In 1993, Alving et al. [5] were the first to employ a chemiluminescence analyzer to find that the concentration of oral expiratory NO in asthma patients was higher than that in healthy people, but the NO values measured by different studies in asthma patients varied greatly. To this end, in 1997, Silkoff et al. [6] tested the FeNO values of 9 different expiratory flows, and found that the FeNO values in the exhaled breath would enter a plateau after a certain period of expiratory time, that is, NO plateau concentration (NO_PLA_), and found that the FeNO values were related to the corresponding expiratory flows. In 1998, Tsoukias and George [7] published a kinetic model of convective diffusion of lung NO through exhaled air, a two‐chamber model, and verified the reliability of the model by comparing FeNO values at different expiratory flows.

#### 3.1.2 Kinetic Principles of Flow Dependence

Based on the principle of flow dependence of FeNO measurement, the 1999 ATS technical standards recommend an expiratory flow rate of 50 mL/s as the standard for measuring FeNO [8]. In 2005, ATS/ERS expanded the technical standards to include FnNO, noting that the measurement of FnNO also showed a negative exponential correlation with nasal airflow [4]. The kinetic principle is that exhaled NO primarily originates from the epithelial cells of the airways and nasal cavity and diffuses into the exhaled breath. As flow rate increases, although NO output increases due to the acceleration of convective diffusion, the total volume of exhaled air increases more obviously, which eventually leads to the decrease of FeNO and FnNO concentration.

#### 3.1.3 Presentation of FeNO_200_

In 2014, researchers proposed that FeNO_200_ was positively correlated with the concentration of NO in alveolar tissues [9], which could be used to indicate the level of NO in the alveoli and surrounding small airways. By measuring FeNO at 50 mL/s and 200 mL/s flows, this method can differentiate between central and peripheral airway inflammation.

### 3.2 Q3: What Detection Parameters Can Be Obtained Through the FeNO Analyzer and What Is the Clinical Significance?

**C3**: The FeNO analyzer can detect FeNO and FnNO, and the two‐chamber lung model can calculate CaNO, but also derive other relevant parameters, such as NO produced by inflammatory tissues in the large airway (CawNO), the diffusion rate of NO from the production site to the airflow body (DawNO), the total NO flux in the airway chamber (JawNO), the NO clearance rate (*V̇*_*NO*_) and the expiratory flow rate (*V̇*_*E*_). These parameters can help clinicians better understand the metabolism and transport processes of NO in tissues.

The evidence is summarized and interpreted below.

#### 3.2.1 Parameters Detected and Derived by the FeNO Analyzer

The parameters detected and derived by the FeNO analyzer are shown in Table 1.

**TABLE 1:** Parameters detected and derived by the FeNO analyzer [3, 10].

Detection or derivation	NO parameter (unit)	Description and significance
Detection	FeNO (ppb)	Oral expiratory NO body integral rate, correlated with *V̇*
		FeNO measured at 50 mL/s was labeled FeNO_50_ and contained small airways, mainly reflecting large airway inflammation
		FeNO measured at 200 mL/s was labeled FeNO_200_ and mainly reflected small airway inflammation
	FnNO (ppb)	Nasal expiratory NO body integral rate, correlated with *V̇*
		FnNO measured at 5 mL/s was labeled FnNO_5_
		FnNO measured at 10 mL/s was labeled FnNO_10_
		FnNO reflects inflammation of the upper airway predominantly in the nasal cavity and sinuses
Derivation	CawNO (ppb)	NO produced by bronchial or large airway tissues, independent of *V̇*
		A true indicator of inflammation in the large airways
	DawNO (mL/s)	Diffusion coefficient of NO from airway epithelium into exhaled airflow
	*V̇*_*E*_ (mL/s)	Expiratory flow
	CaNO (ppb)	Concentration (ppb) of oral exhaled NO calculated by the model formula reflects inflammation of the alveoli or surrounding small airways
	JawNO (nL/s)	The airway NO produces diffuse flux
		JawNO = (CawNO‐CaNO) × DawNO, if CaNO is 0, it is equal to the theoretical maximum flux, JawNO = CawNO × DawNO
		JawNO is highly correlated with FeNO_50_ and is also an indicator of inflammation in the large airways
	*V̇*_*NO*_ (pL/s or nL/s)	NO clearance rate

*Note:* FeNO is exhaled nitric oxide; *V̇* is the flow rate; FeNO_50_ is 50 mL/s flow rate and oral exhaled nitric oxide; FeNO_200_ is 200 mL/s flow rate and oral exhaled nitric oxide; FnNO is nasal exhaled nitric oxide; FnNO_5_ is nasal exhaled nitric oxide at 5 mL/s flow; FnNO_10_ is nasal exhaled nitric oxide at a flow rate of 10 mL/s; CawNO is the NO produced by the inflammatory tissues of the large airways; DawNO is the diffusion rate of NO from the generating site to the main body of the airflow; *V̇*_*E*_ is the expiratory flow; CaNO is alveolar exhaled nitric oxide; JawNO is the total airway chamber NO flux; *V̇*_*NO*_ is the NO clearance rate; ppb is 10^−9^ mol/mol.

#### 3.2.2 Clinical Significances of NO Multiparametric Exploration

Table 1 shows that the true indicators of inflammation are NO produced by the airways and alveolar tissue, specifically CawNO and CaNO, not FeNO. FeNO depends on three factors: CawNO and CaNO, DawNO, and *V̇E*. The simplified formula derived from the dual chamber model equation shows the relationship between them.

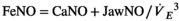

$$\text{JawNO}=\left(\text{CawNO}-\text{CaNO}\right)\times {\text{DawNO}}^{3}$$

Notably, the clinical use of FeNO as an indicator of airway inflammation has certain limitations. For example, in critically ill patients with airway diseases, although the patient's CawNO is elevated, excessive mucus secretion forms a diffusion barrier, causing a decrease in DawNO, which in turn leads to a decrease in FeNO, and the FeNO value at this time does not reflect the true level of airway inflammation. Therefore, the 2011 ERS technical standards propose multi‐parameter NO determination, including FeNO, CaNO, CawNO, and DawNO, to enable in‐depth exploration of the clinical significance of NO through multi‐parameter and multi‐dimensional studies [10].

## 4 FeNO Detection Methods

### 4.1 Q4: What Are the Detection Methods of FeNO? What Is the Recommended Standardized Testing Method?

**C4:** The detection methods of FeNO include online testing, offline testing, and tidal breathing testing. Online testing is recommended as a standardized testing method.

The evidence is summarized and interpreted below.

In 2005, ATS/ERS published a standardized process for FeNO testing, including online testing, offline testing, and moisture testing [4]. This standardized process is widely used today, with the online test recommended by international guidelines and approved by the U.S. Food and Drug Administration (FDA). Due to the difficulty of quality control of offline testing and moisture testing, these methods have not yet been approved by the FDA for clinical practice application.

#### 4.1.1 Standard Procedure for Online Inspection

(1) Inhalation of gas: During testing, the subject should first exhale deeply to empty the lungs as completely as possible, and then inhale NO‐free air (< 5 ppb NO). For this purpose, both the equipment and breathing handle must be equipped with NO scrubbers to avoid environmental NO contamination. (2) Inspiratory stage: inhale with a filter in the mouth for 2~3 s to reach or approach the total lung volume, and then exhale immediately to avoid the influence of breath‐holding on FeNO value. (3) Exhalation phase: Completed in one breath, the FeNO standardized test ensures reproducibility through the following two key factors [4]. ① Exclusion of nasal NO: Usually the subject exhales against resistance, and the expiratory pressure is 5~20 cmH_2_O (1 cmH_2_O = 0.098 kPa), ensuring that the soft palate is closed to avoid nasal NO contamination [4]. ② Standardized expiratory flow: The expiratory flow rate is maintained at 45~55 mL/s to achieve a stable NO concentration platform and meet the detection requirements of patient comfort, detection sensitivity and reproducibility [4]. (4) Exhalation time: Slow and steady exhalation for 10 s (≥ 4 s for children ≤ 12 years, and at least 6 s for children over 12 years old and adults). Since NO has an early peak and peak washout period during the initial 6–7 s of exhalation, followed by a stable plateau in the later phase, the mean value within a 3‐s window of the plateau period is used for interpretation.

#### 4.1.2 Offline Testing Procedure

Offline detection refers to the collection of exhaled breath into a suitable container for later analysis. (1) Inhalation of gas: In order to avoid environmental NO contamination, it is necessary to achieve this by placing a NO scrubber in the inhalation device or inhaling air from a reservoir that does not contain NO gas [4]. (2) Exhalation phase: After inhaling, exhale slowly and forcefully, making sure that the soft palate is closed, and collect exhaled breath into a special storage bag [4]. The offline test needs to be completed with multiple exhalations, and the sample collection of the offline test needs to exclude the initial 150~200 mL airway invalid lumen gas and maintain a constant expiratory flow, which is crucial for the quality control of the offline test, the standardization of the measurement, and the comparability with the online test [4]. (3) Gas storage and analysis: The reservoir must be NO‐inert and relatively impermeable. Suitable materials include polyvinyl fluoride film and polyester film. Offline detection of the sample gas should be analyzed and determined within 48 h [4]. (4) Clinical application: for online and offline detection, even if the same flow rate is used, the results and cut‐off point values are not interchangeable [4]. Offline testing is not methodologically perfect, and further clinical research is needed [10].

#### 4.1.3 Tidal Breathing Detection

First, the mask is placed on the mouth and nose of the infant who is kept quiet, and the child inhales air containing NO < 4 ppb through the air filter and breathes calmly for 30~60 s. The collection bag is attached to the expiratory port of the tidal breathing sampler to gather the exhaled gas from the child. The collected gas is fed into the FeNO analyzer for detection. The exhaled air collected by this method is a mixture of mouth and nose, and given that infants' paranasal sinuses are undeveloped, their influence on the collected gas is negligible. Therefore, the sampled gas can be considered to originate primarily from the large airways. However, as children grow, their paranasal sinuses develop progressively. When tidal breathing analysis is applied to older children, the influence of nasal NO cannot be entirely excluded, compromising result interpretation. At present, there is no unified clinical cut‐off point for this population, and the influence of age needs to be considered in both children and adolescents, and the FeNO cut‐off point value decreases by 1 ppb for every 1 year of age. Methodologically, tidal breathing measurement remains imperfect, and its clinical implementation requires further standardization [4].

### 4.2 Q5: What Are the Detection Methods for FnNO? Which One Is Recommended for Adults? What Is the Usual Nasal Expiratory Flow for Measuring FnNO?

**C5:** There are three detection methods for FnNO: exhalation‑against‑resistance method, breath‐hold and tidal breathing, and the exhalation‑against‑resistance method method is the first recommended for adults. When measuring FnNO, the nasal expiratory flow can be selected at 10 mL/s and expressed as FnNO_10_.

The evidence is summarized and interpreted below.

The 2023 ERS technical standard states that the standard technique for the detection of FnNO is chemiluminescence or electrochemical method, and both methods have been approved for clinical use in Europe [1, 2], and the specific detection methods are as follows:

#### 4.2.1 Exhalation‑Against‑Resistance Method

This method is applicable to children aged five and older as well as adults, and is recommended as the preferred approach for FnNO testing. Subjects select an appropriately sized olive head to be fully inserted into the selected nostril, inhale with a full air, and exhale slowly with their mouth toward the flow restrictor (e.g., breathing handle, blow roll toy) to ensure that the soft palate is closed. Recommended sample flow rate is 0.3~0.33 L/min for 10 s, and the repeatability is better. The assay should be performed in software mode, which allows the technician to view the FnNO concentration profile in real time and manually determine the preferred platform value when the test is completed [1, 2]. The FnNO platform should be ≥ 3 s with a < 10% variation over the measurement range.

#### 4.2.2 Breath‐Holding Method

The breath‐holding method is an alternative to the resistance method. The procedure is as follows: The subject inhales deeply to total lung capacity, holds the breath to close the glottis and soft palate via Valsalva maneuver.

#### 4.2.3 Tidal Breathing Method

The tidal breathing method is used in infants < 5 years of age, and an olive head is inserted into the child's nostril, and the tidal breathing can be stabilized with the mouth open or closed for ≥ 30 s. However, as the children cannot close their soft palate during exhalation, the collected gas represented a mixture of nasal and oral air, resulting in greater variability in the measured results. This method has a recommended cut‐off point value for children aged 2~5 years, but it is not diagnostic for the FnNO test results of healthy infants < 12 months old, and is only used as a research tool [1].

### 4.3 Q6: What CaNO Calculation Methods Exist? As a Calculated Value, Is CaNO Suitable for Clinical Applications?

**C6:** CaNO can be calculated by three methods derived from the two‐chamber model of the lungs (nonlinear model, linear model, and simplified linear model). As a calculated value, the CaNO error is difficult to avoid, and the CaNO value combined with FeNO_200_ obtained based on the linear model has certain reference significance for the evaluation of small airway inflammation, but the specific cut‐off value remains undetermined.

The evidence is summarized and explained below.

#### 4.3.1 Method 1: Nonlinear Model

The nonlinear model requires the subjects to perform FeNO measurements at least twice at low (< 20 mL/s), medium (100 mL/s), high (350 mL/s, or even 400 mL/s) flow rates, and the average flow rate and average concentration are used to calculate CaNO. The advantage of this method lies in its relatively small model error. However, several limitations must be noted: (1) measurement variability increases significantly with 6 repeated determinations at 3 flow rates; (2) the requirement for model‐based back‐calculation of FeNO_50_ for comparative validation introduces procedural complexity; (3) clinical application is constrained as many adults and children with airway diseases cannot reliably perform high‐flow FeNO maneuvers.

#### 4.3.2 Method 2: Linear Model

The CaNO calculated by the linear model needs to be tested twice at each of the three flow rates of 100~400 mL/s, and the average flow rate and average concentration are used to calculate CaNO. The disadvantage of this model is that the measurement error caused by the six measurements of three different flow rates is large. At the same time, because the model does not cover FeNO_50_, the calculation results cannot be verified. In addition, the difficulty of completing the determination of high‐flow FeNO also limits the usage of this method.

#### 4.3.3 Method 3: Simplified Linear Model

The simplified linear model uses FeNO_50_ and FeNO_200_ measured by double flow rates, and the CaNO is calculated using the correction factor. A 2014 study from the United Kingdom showed that FeNO_200_ was positively correlated with CaNO for multi‐flow calculations [9]. The 2021 *Chinese Expert Consensus on Exhaled Nitric Oxide Detection and Its Application in the Diagnosis and Treatment of Airway Diseases* pointed out that linear model and nonlinear models have certain difficulties in actual clinical operation, so CaNO can be calculated according to the FeNO_50_ and FeNO_200_ measurement results at 50 mL/s and 200 mL/s flow rates through a simplified linear model formula for the assessment of small airway inflammation. The simplified linear model is relatively simple to calculate CaNO, but it still cannot completely replace the linear model and the nonlinear model, and its effectiveness needs to be continuously verified in clinical practice.

## 5 Quality Control for FeNO Testing

### 5.1 Q7: What Factors Should Be Avoided Before FeNO Testing (Pre‐Test Quality Control)?

**C7:** Before FeNO detection, care should be taken to minimize environmental interference; additionally, multiple confounding factors—including age, gender, diet, smoking, medications, infections, and other factors—may affect the measurement. FeNO testing should be completed before routine pulmonary function testing to ensure the accuracy.

The evidence is summarized and explained below.

#### 5.1.1 Environmental Factors

NO sensors require stable environmental conditions. Each manufacturer specifies acceptable ranges for temperature, humidity, and barometric pressure in the instrument manual. New sensors require a 3‐h stabilization period after installation; this duration should be extended if environmental conditions change significantly. High ambient NO levels (from pollution, disinfectants, or UV exposure) may affect measurements. Hypoxic conditions at high altitudes may yield falsely low FeNO values. Such conditions should be documented in the report [4].

#### 5.1.2 Age/Sex

Increased age in adults may lead to decreased alveolar NO absorption; FeNO levels are slightly elevated in males > 60 years and females > 45 years [11].

#### 5.1.3 Diet

Dietary nitrites can reduce L‐arginine to promote the production of NO, and the acidic environment will enhance this reaction, leading to elevated FeNO levels. Foods such as caffeine, sugar (fructose), ethanol, and high‐fat meals transiently alter FeNO levels by modulating NO synthase activity. Patients should avoid these substances for ≥ 1 h before testing [12].

#### 5.1.4 Smoking

Chronic smoking reduces FeNO levels. Acute effects are inconsistent. Abstinence from smoking for ≥ 1 h before testing is recommended [12, 13].

#### 5.1.5 Medications

Drugs can bias test results by influencing NO production or apparent levels. Inhaled or oral corticosteroids, type 2 inflammation‐associated monoclonal antibodies, NO synthase inhibitors, leukotriene receptor antagonists, and antihistamines cause FeNO to decrease; NO donor drugs (e.g., nitroglycerin or L‐arginine) and bronchodilators can cause FeNO to be elevated. In addition, nasal medications such as nasal decongestants, nasal steroids, and vasodilators may alter FnNO results. Document all medications before testing [4, 12].

#### 5.1.6 Exercise and Pulmonary Function Tests

The effects of exercise on FeNO have been reported inconsistently, and it is recommended to avoid strenuous exercise for 1 h before testing [12]. Forced expiratory action will reduce the FeNO value and should be avoided as much as possible before testing. Spirometry reduces FeNO for approximately 1 h post‐test. Perform FeNO testing first [14].

Although duplicate measurements are recommended whenever feasible, this approach may not always be practical in busy clinical settings. In such circumstances, the following alternative quality‐control measures may be adopted:

1. Verification of stable expiratory flow throughout the maneuver;
2. Confirmation of an acceptable NO plateau lasting at least 3 s;
3. Daily instrument self‐calibration according to manufacturer specifications;
4. Routine biological control testing using healthy volunteers or internal quality‐control procedures;
5. Immediate repetition only when substantial flow instability or irregular breathing patterns are observed.

These measures may improve reliability while maintaining workflow efficiency in routine clinical practice.

### 5.2 Q8: What Sampling Irregularities Affect FeNO Accuracy?

**C8:** In FeNO testing, some technical errors may compromise FeNO measurements: incomplete pre‐sampling exhalation, suboptimal inhalation volume, and air leakage during sampling will lead to inaccurate measurement. Subjects should obtain ≥ 2 reproducible measurements (≤ 10% variation) with 30‐s rest intervals between maneuvers.

The evidence is summarized and explained in the following sections.

#### 5.2.1 Exhalation Maneuvers Before Sampling

Before sampling, the residual air in the lungs should be fully exhaled to avoid environmental NO interference. However, some guidelines also state that this step does not affect the test result, and exhaling before inhalation is not necessary [3].

#### 5.2.2 Inadequate Inhalation

The subjects must seal lips tightly around the filter mouthpiece, and inhale through the mouth for 2~3 s to close to the total lung capacity. In accordance with the ERS technical standards, to improve subject comfort, the procedure is adjusted to require a deep mouth inhalation [3].

#### 5.2.3 Air Leakage During the Sampling Process

Air leakage during sampling should be avoided, which can easily cause low FeNO values. Before testing, the operator should verify the integrity of the gas path connection of the instrument and equipment, confirm the subject maintains leak‐free mouthpiece‐lip seal during exhalation.

### 5.3 Q9: How Is the FeNO Analyzer Routinely Maintained and Calibrated? What Attention Should Be Paid to Daily Infection Control?

**C9:** The core components of the chemiluminescence analyzer are inspected annually; standard gases are used for monthly calibration; daily zero calibration is performed. The electrochemical analyzer utilizes pre‐calibrated sensors that generally do not require recalibration but may be equipped with optional calibration modules. For daily contamination control: After using disposable filter tips, the handle and surrounding areas must be cleaned by wiping with a cloth dampened with water, mild soap solution, or sodium hypochlorite solution < 1.5%. Ethanol‐based disinfection is contraindicated for all instruments due to potential sensor sensitivity impairment.

The evidence is summarized and explained follows.

#### 5.3.1 Routine Maintenance and Calibration of Chemiluminescence Analyzers

The instrument needs regular calibration, with calibration frequency increased under unstable conditions. The calibration concentration includes zero calibration and span calibration using standard gas covering the expected sample measurement range [4], according to ERS technical standards, the required standard gas concentration is 2000 ppb, along with annual inspections of the chemical reaction converter, ozone generator, and associated peripheral components [3].

#### 5.3.2 Calibration of Electrochemical Analyzers

The electrochemical analyzer and NO sensor undergo factory validation and calibration prior to distribution. The NO sensor functions as a replaceable reagent package that guarantees accuracy and repeatability under specified conditions without requiring user calibration. In practice, instrument performance may drift over time or following sensor replacement [15, 16]. When equipped with a standard gas calibration module, user calibration is permitted when necessary.

## 6 Clinical Application Value of FeNO

### 6.1 Q10: Is the Value of FeNO Clinically Diagnostic? What Is the Least Cut‐Off Point Value That Hints?

**C10:** FeNO can be recognized as a marker of type 2 airway inflammation. FeNO levels of 25~50 ppb indicate suspicious type 2 airway inflammation, and > 50 ppb indicates type 2 airway inflammation. For patients with suspected asthma and FeNO of 25~50 ppb, when combined with other clinical indicators, asthma can be highly suspected. FeNO > 50 ppb with clinical evidence of asthma supports the diagnosis of asthma. In patients with chronic or subacute cough, the diagnosis of corticosteroids responsive cough (CRC) can be made when FeNO > 25 ppb and clinical features of CRC are present. In patients with confirmed asthma, FeNO can also assess asthma control levels, guide treatment regimen adjustments, predict acute exacerbation risk and predict the efficacy of some biologic therapies when used in combination with other indicators.

The evidence is summarized and explained as follows.

FeNO tends to be higher in healthy adults than in children (< 12 years). According to the 2011 ATS clinical guidelines, normal FeNO in healthy adults is < 25 ppb (20 ppb for children). In adults and children with suspected asthma symptoms, FeNO <25 ppb in adults (< 20 ppb in children) indicates absence of eosinophilic airway inflammation; FeNO > 50 ppb in adults (> 35 ppb in children) suggests eosinophilic airway inflammation. When correlated with compatible clinical features of asthma, these values support asthma diagnosis; “For adults with FeNO levels of 25–50 ppb (20–35 ppb in children), comprehensive clinical evaluation is required to determine an asthma diagnosis [10].

Numerous European and American clinical studies have found that treatment‐naïve adults newly diagnosed with asthma who exhibit intermediate FeNO levels (20–25 ppb) show similar responsiveness to inhaled corticosteroids (ICS) as those with high FeNO levels. Therefore, the 2014 European expert consensus updated the previous ATS guidelines by redefining eosinophilic airway inflammation as type 2 airway inflammation. It recommends that in suspected asthma cases, FeNO > 25 ppb suggests probable type 2 airway inflammation, which‐when combined with other clinical indicators‐may support an asthma diagnosis (Table 2) [13]. With the advent of biologic therapies, clinical determination of type 2 airway inflammation has become essential. Currently, FeNO, peripheral blood eosinophils, sputum eosinophils, and immunoglobulin E are commonly used biomarkers to determine type 2 airway inflammation.

**TABLE 2:** Comparison table of FeNO cut‐off values for diagnosing types of airway inflammation commonly used in clinical practice.

Types of airway inflammation	FeNO cut‐off values (ppb)	
	> 12 years old	≤ 12 years old
Non‐type 2 inflammation	< 20~25^a^	< 15~20^a^
Suspicious type 2 inflammation	20/25~50	15/20~35
Type 2 inflammation	> 50	> 35

*Note:* FeNO is exhaled nitric oxide.

^a^ To determine the cut‐off point value according to the patient's gender, age and height; ppb is 10^−9^ mol/mol.

ICS is the cornerstone of asthma treatment, and FeNO predicts patient responsiveness to ICS treatment. One study comparing physicians' assessment accuracy of airway inflammation (based on symptoms, signs, and lung function) found < 50% accuracy without FeNO values [17]. ICS therapy response rates significantly increase when FeNO > 25 ppb, suggesting corticosteroid responsiveness and supporting ICS initiation [18].

Among common causes of chronic cough, cough variant asthma (CVA), eosinophilic tracheitis, and allergic cough—collectively termed corticosteroid‐responsive cough (CRC)—respond well to ICS. CRC patients exhibit significantly higher FeNO levels than non‐CRC patients. FeNO > 31.5 ppb demonstrates high specificity but moderate sensitivity for CRC diagnosis, whereas FeNO < 22.5 ppb makes CRC unlikely. The 2023 British Thoracic Society Consensus recommends that FeNO > 25 ppb in chronic/subacute cough patients indicates eosinophilic airway inflammation, indicating a high CRC probability and predicting favorable ICS response [18].

As a type 2 airway inflammation biomarker, elevated FeNO levels associate with increased peripheral blood/sputum eosinophils and asthma exacerbations. FeNO ≥25 ppb shows 65% sensitivity for predicting ≥ 2 annual exacerbations [19]. Combining multiple parameters (symptoms, eosinophils, lung function) improves exacerbation prediction reliability. A meta‐analysis (35 studies) showed high FeNO levels are associate with accelerated lung function decline in severe asthma patients, despite optimal treatment [20]. FeNO‐guided strategies reduce exacerbation frequency [21].

FeNO also predicts response to biologics. Omalizumab shows greater efficacy (53% vs. 16% exacerbation reduction) when baseline FeNO ≥ 24 ppb [22]. As its distinctive eosinophil‐depleting mechanism, Benralizumab demonstrates consistent efficacy (80% exacerbation reduction) across all FeNO levels [23]. Tezelumab shows 50% exacerbation reduction when FeNO < 25 ppb, improving to 75% when FeNO ≥25 ppb, and its application is not limited to FeNO values [24]. Dupilumab achieves 60%–70% exacerbation reduction when FeNO ≥ 25 ppb, with parallel improvements in pulmonary function, symptomatic control, and quality‐of‐life metrics [25, 26, 27].

### 6.2 Q11: Does CaNO Value Have Adjunct Diagnostic Value? What Is the Cut‐Off Value?

C11: CaNO serves as a small airway/alveolar inflammation biomarker with clinical utility for chronic respiratory disease evaluation, though standardized cutoffs remain undefined.

The evidence is summarized and explained as follows.

CaNO reflects distal airway inflammation/obstruction [28]. A 2019 meta‐analysis of 33 studies found elevated NO parameters in asthma patients, though CaNO measurements showed significant heterogeneity [28]. In symptomatic asthma, CaNO positively correlates with blood eosinophils and negatively with small airway function (forced expiratory flow at 50% of FVC exhaled (FEF_50_)) and forced expiratory flow at 75% of FVC exhaled (FEF_75_), suggesting small airway dysfunction [29]. For asthma patients with small airway inflammation, CaNO values decreased after ICS, further demonstrating the value of CaNO as an indicator of small airway inflammation [30]. However, lack of standardized methods limits widespread application, requiring further multicenter studies.

### 6.3 Q12: Does FnNO Has Adjunct Diagnostic Value? What Are the Cutoff Values?

**C12**: Normal FnNO ranges from 250–500 ppb in healthy individuals. Allergic rhinitis patients show elevated FnNO correlating with severity, whereas non‐allergic rhinitis patients show normal levels. Chronic sinusitis patients demonstrate reduced FnNO. FeNO for Cystic fibrosis (CF) patients ranges 70–300 ppb (lower with nasal polyps). FnNO < 77 ppb supports primary ciliary dyskinesia (PCD) diagnosis.

Here is the evidence summary and interpretation:

Nasal NO derives from sinus epithelium via inducible NO synthase. Allergic rhinitis shows FnNO elevation proportional to inflammation severity, unlike non‐allergic rhinitis [31]. Chronic sinusitis reduces FnNO due to sinus ostia obstruction preventing NO release, though eosinophilic inflammation may increase levels—requiring comprehensive evaluation [32].

CF is a disease caused by mutations in the CFTR gene. CFTR mutations cause dysregulation of salt and fluid secretion in the respiratory tract, resulting in viscous respiratory sputum and secondary bronchiectasis. Destruction of bronchial structure and abnormal mucociliary function can lead to NO production and transport disorders in CF patients, usually CF patients with FnNO fluctuating at 70~300 ppb, and the FnNO level of CF patients with nasal polyps is significantly lower than that of CF patients without nasal polyps [33].

PCD is a rare disease affecting the upper respiratory tract (anosmia, sinusitis, chronic tonsillitis), lower respiratory tract (bronchiectasis), and male infertility (impaired sperm swing). Diagnosis is usually made by evaluation of respiratory ciliary ultrastructure and/or genetic testing. Clinically in patients with a high suspicion of PCD, NO testing is a sensitive and specific screening modality for PCD. For Children aged 5~17 years, after excluding CF, FnNO < 127 ppb shows 98% sensitivity and 99% specificity for PCD [34]. The European Society of Rhinology guidelines state that in patients with clinical symptoms of PCD, a FnNO < 77 ppb indicates the diagnosis of PCD, and its diagnostic reliability is comparable to that of electron microscopy or genetic testing [35].

### 6.4 Q13: What Is FeNO Multi‐Index Testing's Clinical Value?

**C13**: Combining FeNO with other tests (lung function, symptoms) improves diagnostic accuracy. These include FeNO plus small/large airway parameters, FeNO plus symptom scores for treatment adjustment and combined FnNO/FeNO/CaNO for assessing upper/lower/small airway inflammation. However, clinical studies on combined testing in chronic airway diseases remain limited.

The following is the evidence summary and interpretation:

CVA is a common cause of chronic cough, and in the diagnosis of CVA, when there is no clear basis for variable airflow limitation or bronchial provocation test cannot be performed, combining FEF25–75% pred (< 78.5%) with FeNO (> 43 ppb) predicts positive bronchoprovocation [36]. For typical asthma symptoms with FEV1% pred ≥ 80%, ΔFEV1% pred (> 3.5%) plus FeNO (> 33 ppb) predicts ICS/formoterol response [37]. The Asthma Control Questionnaire (ACQ) combined with FeNO level (> 29 ppb) was employed to guide escalation/de‐escalation of treatment. ACQ plus FeNO (> 29 ppb) guidance reduces exacerbations (25% vs. 41%) and ICS doses versus ACQ alone [38].

At present, multiple brands of FeNO analyzers can complete the joint inspection of FeNO, FnNO and CaNO simultaneously. FeNO combined with FnNO can evaluate the type of inflammation in both large and upper airways, whereas FeNO combined with CaNO can determine the type of inflammation in large airways and alveoli/small airways. Based on the principle of “one airway, one disease”, comprehensive characterization of airway inflammation is essential for targeted therapeutic strategies. However, there are relatively few series studies on the value of NO multiplex testing in the diagnosis and treatment of chronic airway diseases [39].

## 7 Standardized Formats for FeNO Test Reports

**Q14:** What is the minimum information contained in the FeNO test report to ensure clinical interpretation? Is it necessary to list the detection curves, normal values, and anomaly ranges on the report?

**C14**: The FeNO test report should consist of at least 3 parts. (1) Basic information: including the general condition of the subject, brief relevant medical history, and testing time, etc. (2) Test information: including measurement parameters, measured values, historical trend charts of measured values, flow‐time curves, and concentration‐time curves, etc. (3) Clinical reference information: including normal values, abnormal ranges and corresponding significance.

Here is the evidence summary and interpretation.

### 7.1 Report Template

**FIGURE 1:**
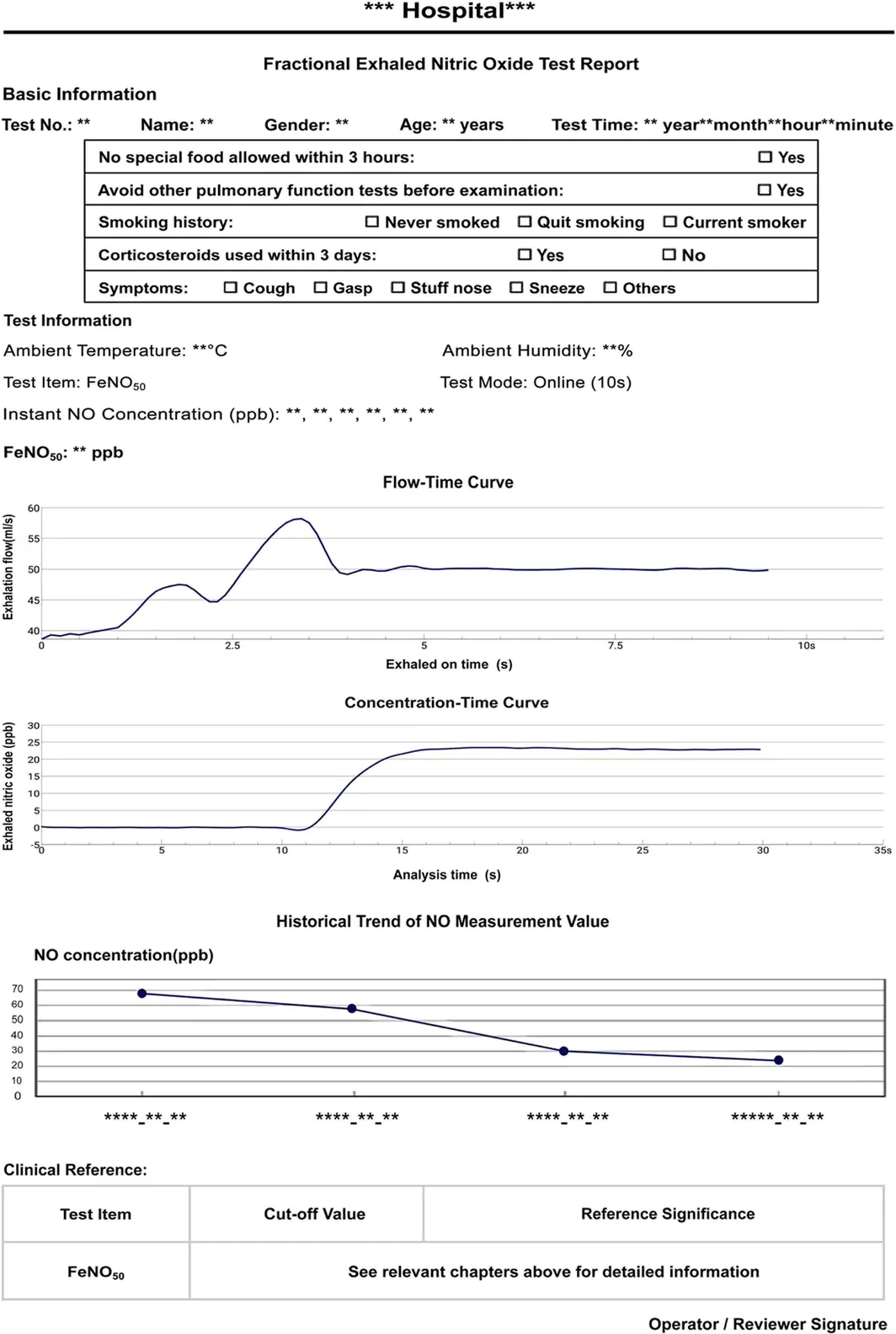
FeNO_50_ test report. Note: FeNO_50_ is 50 mL/s flow rate with oral exhaled NO; ppb is 10^−9^ mol/mol.

A FeNO_50_ report template based on the ATS clinical guidelines is provided (see Figure 1).

### 7.2 Significance of Real‐Time Display of the Test Curve

According to the ATS/ERS technical standard, the FeNO analyzer performs continuous real‐time sampling, measurement, and display of both expiratory flow and NO concentration as functions of expiratory time [4]. On the one hand, real‐time monitoring ensures measurement reproducibility by verifying consistent expiratory flow rates. On the other hand, the real‐time display of NO concentration helps to analyze the stability of the detected signal. Electrochemical analyzers are typically designed with an internal temporary gas chamber, which features a coiled or channel‐like configuration. This design ensures precise timing characteristics for both sample collection and detection. Through the real‐time waveform display of flow rate and concentration, the quality control of FeNO measurement can be comprehensively assessed, including evaluation of repeatability and stability.

## 8 Summary and Outlook

With the rapid development of NO measurement technology, FeNO, CaNO and FnNO have been implemented in clinical practice, which provides significant assistance for clinical diagnosis and treatment (A summary of the 14 consensus questions above and their corresponding recommendations is provided in Table S1, offering a quick reference for clinicians and researchers). However, there are still some issues that require further research, such as the frequency of NO detection, clinical significance and reference values of tidal breathing NO measurement, cut‐off values, of FeNO and CaNO in different respiratory diseases, and clinical value of FnNO in upper airway diseases. As to whether FeNO needs to be repeated, according to the 2005 ATS/ERS technical standards [4], subjects should perform repeated exhalations to obtain at least 2 FeNO values with < 10% difference. FeNO is calculated as the average of the two values. According to the 2017 ERS technical standards [3], if the variation between two consecutive exhaled NO measurements exceeds 10%, an additional exhalation should be performed [3]. However, due to influences from different instruments, hospitals and other factors, this has not yet been widely adopted in clinical practice. Therefore, we recommend performing duplicate measurements during each FeNO assessment when feasible to ensure reproducible results. In settings where routine repeat testing is impractical, monthly biological control tests should be implemented using trained operators (typically laboratory technicians), and at least 2 tests in the same state, and the variability should be < 10%; if the standards are not met, the accuracy of the instrument should be confirmed.

Although some FeNO studies have been conducted, China still lacks diagnostic criteria including normal values and reference ranges from large‐scale multicenter studies. In the future, more and more comprehensive studies are needed to validate FeNO's diagnostic utility across respiratory diseases, establish its role in treatment efficacy monitoring, determine its prognostic value in chronic airway diseases and develop standardized protocols for Chinese clinical practices.

## 9 Knowledge Gaps and Future Research Directions

Despite substantial progress in the standardization of FeNO measurement and its integration into clinical algorithms for type 2 airway inflammation, several unresolved issues persist across technical, physiological, and translational domains.

Current ATS/ERS cut‐offs are predominantly derived from adult Caucasian populations and children aged 6–12 years, with limited data for older adults (≥ 65 years) across sex‐specific strata. Normative reference intervals remain inadequately defined for key demographic subgroups. The clinical utility of CaNO and other derived nitric oxide parameters requires further validation in large prospective studies. Moreover, standardization of FeNO measurements across different analyzer technologies remains an ongoing challenge. In addition, the implementation of simplified yet reliable quality control protocols suitable for high‐throughput clinical settings warrants further investigation. Current recommendations including daily zero calibration, monthly span checks, and stringent pre‐test restriction regimens are well‐validated for research environments but pose logistical barriers in primary care and busy outpatient clinics. Pragmatic alternatives, such as algorithm‐based anomaly detection or embedded biological control procedures, require prospective evaluation.

Future research should prioritize (1) large‐scale, multicenter population‐based studies to derive ethnicity‐ and age‐stratified reference equations; (2) head‐to‐head method‐comparison trials using standardized reference materials to establish interchangeability criteria; (3) prospective longitudinal cohorts evaluating derived NO parameters against hard clinical endpoints (e.g., exacerbation rate, lung‐function decline, histologic inflammation); and (4) implementation science research to validate user‐friendly, cost‐effective quality‐assurance workflows that preserve measurement integrity while reducing operational burden.

Ultimately, the integration of FeNO‐based metrics into precision medicine frameworks for airway diseases will require harmonized technical standards, robust real‐world evidence, and accessible decision‐support tools for clinicians.

## Author Contributions

Yuanlin Song and Zhihong Chen conceived and supervised the project. Li Li, Jian Guo, Yan Zhou, Ying Gong, and Zhihong Chen participated in literature review, evidence appraisal, and recommendation development. Yulan Qu updated the literature, prepared the English manuscript, and coordinated manuscript revision. All authors contributed to interpretation of evidence, critical revision of the manuscript, and approved the final version.

## Funding

This work was supported by the National Key Research and Development Program of China (2020YFC2003700). The study followed the Practice Guideline Registration for Transparency (PREPARE‐2024CN685).

Note: This is a republication of the full translated version of the “Chinese expert consensus on the testing process and clinical application of fractional exhaled nitric oxide detector (2025)” [40], authorized for publication with permission from the original publishing journal, International Journal of Respiratory Medicine.

## Conflicts of Interest

The authors declare no conflicts of interest.

## Supporting information


**Figure S1:** PRISMA flow diagram of the literature search and study selection process.

**Table S1:** Summary of Questions and Recommendations.

## Data Availability

Data sharing not applicable to this article as no datasets were generated or analysed during the current study.
