# Supplementary material for: Chinese Expert Consensus on the Testing Process, Quality Control and Clinical Application of Fractional Exhaled Nitric Oxide (2025)
Source: Clin Respir J. 2026 Sep 18;20(9):e70222. doi: 10.1111/crj.70222 (PMC13588943; doi:10.1111/crj.70222)
Supplement: Supplementary file 1 [file CRJ-20-e70222-s002.docx]

**Table S1. Summary of Questions and Recommendations**

| **Question (Q)** | **Summary of Recommendation / Key Point** |
| --- | --- |
| **Q1: Detection Principles & Inter-Instrument Consistency** | **Principles:** Chemiluminescence (reference standard, high sensitivity) vs. electrochemical (portable, widely used clinically).  **Consistency:** Both adhere to identical ATS/ERS technical standards; however, susceptibility to distinct interferents and divergent technical parameters preclude full interchangeability. |
| **Q2: Flow Dependence** | **Strictly flow-dependent.**  • Standard flow 50 ml/s (FeNO50) reflects large-airway inflammation.  • Flow 200 ml/s (FeNO200) reflects small-airway/alveolar inflammation.  • Inverse relationship: Higher expiratory flow yields lower FeNO values. |
| **Q3: Measurable & Derived Parameters** | **Directly measured:**  FeNO (airway inflammation), FnNO (upper airway inflammation).  **Derived:**  • CaNO (alveolar/small-airway inflammation).  •CawNO (true large-airway inflammatory output).  Utility: Enables differentiation between central and peripheral airway inflammatory sources. |
| **Q4: Standardized Testing Method** | **First-line recommendation:** On-line testing (FDA-cleared, international standard).  **Alternatives:** Off-line and tidal breathing methods (limited by quality-control challenges; not routinely recommended) |
| **Q5: FnNO Measurement** | **Preferred method for adults:** Resistance method (against expiratory resistance).  Standard flow rate: 10 ml/s (reported as FnNO10). |
| **Q6: CaNO Calculation** | **Common approach:** Simplified linear model (derived from FeNO50 and FeNO200).  **Clinical relevance:** Offers adjunctive information on small-airway inflammation, but no standardized cut-off value has been established. |
| **Q7: Pre-Test Quality Control** | **Key restrictions** (≥1 hour pre-test): Avoid food, smoking, caffeine, alcohol, and strenuous exercise. Perform FeNO before spirometry or other pulmonary function tests.  Document all concomitant medications (e.g., corticosteroids, bronchodilators). |
| **Q8: Sampling Maneuver Errors** | **Common pitfalls:** Inadequate inspiratory volume, air leaks (incomplete lip seal).  **Quality requirement:** Obtain ≥2 reproducible measurements with ≤10% coefficient of variation |
| **Q9: Instrument Maintenance & Infection Control** | **Chemiluminescence:** Requires routine calibration with standard gases.  **Electrochemical:** Pre-calibrated sensors; user calibration generally unnecessary.  **Disinfection:** Alcohol-based agents are contraindicated; use <1.5% sodium hypochlorite or mild soapy water for cleaning. |
| **Q10: Clinical Cut-offs for FeNO** | **Adult cut-offs:**  • < 25 ppb: Non-type 2 inflammation.  • 25–50 ppb: Suspected type 2 inflammation.  • > 50 ppb: Type 2 inflammation; supports asthma diagnosis when combined with compatible clinical features.  **Chronic cough:** FeNO > 25 ppb suggests corticosteroid-responsive cough. |
| **Q11: CaNO Adjunctive Diagnostic Value** | **Value:** Biomarker for small-airway/alveolar inflammation.  **Cut-off:** No unified cut-off value currently defined; interpretation requires integrated clinical assessment. |
| **Q12: FnNO Adjunctive Diagnostic Value** | **Key cut-off:**  **FnNO < 77 ppb** supports diagnosis of primary ciliary dyskinesia (PCD) (high sensitivity/ specificity).  **Allergic rhinitis:** Elevated FnNO; chronic rhinosinusitis: Reduced FnNO. |
| **Q13: Multi-Parameter Combined Testing** | **Utility:** FeNO combined with lung function or symptom scores improves diagnostic accuracy and guides therapy titration.  FeNO + FnNO + CaNO enables comprehensive upper/lower/small-airway inflammation profiling ("one airway, one disease"), though robust clinical outcome data remain limited. |
| **Q14: Essential Report Content** | **Minimum requirements (3 sections):**   - Patient demographics and pertinent history; - Test parameters, numeric values, and **flow-time and concentration-time curves** (critical for quality verification); - Reference ranges, abnormal thresholds, and corresponding clinical interpretations. Curves and reference ranges are mandatory for proper interpretation. |
